# Transcriptomic Analysis of Root Restriction Effects on the Primary Metabolites during Grape Berry Development and Ripening

**DOI:** 10.3390/genes13020281

**Published:** 2022-01-30

**Authors:** Feng Leng, Yue Wang, Jinping Cao, Shiping Wang, Di Wu, Ling Jiang, Xian Li, Jinsong Bao, Naymul Karim, Chongde Sun

**Affiliations:** 1 Laboratory of Fruit Quality Biology, The State Agriculture Ministry Laboratory of Horticultural Plant Growth, Development and Quality Improvement, Zijingang Campus, Zhejiang University, Hangzhou 310058, China; lengfeng.214@163.com (F.L.); fruit@zju.edu.cn (Y.W.); di_wu@zju.edu.cn (D.W.); xianli@zju.edu.cn (X.L.); adesun2006@zju.edu.cn (C.S.); 2 College of Horticulture and Plant Protection, Yangzhou University, Yangzhou 225009, China; 3 School of Agriculture and Biology, Shanghai Jiao Tong University, Shanghai 200240, China; fruit@sjtu.edu.cn; 4 Wujiang Research Institute of Grape, Jinhua 321017, China; jlonly@sina.com; 5 Institute of Nuclear Agricultural Sciences, Zijingang Campus, Zhejiang University, Hangzhou 310058, China; jsbao@zju.edu.cn; 6 Department of Food Science and Nutrition, Zhejiang University, Hangzhou 310058, China; naiemph@zju.edu.cn

**Keywords:** root restriction, primary metabolites, RNA-Seq, grape berry

## Abstract

Root restriction (RR) has been reported to enhance grape berry quality in diverse aspects of grape life. In this study, RR-induced increases in the main primary metabolites in the grape berry and the expression of their related genes were studied at different developmental stages. Mainly the transcriptomic and metabolomic level were analyzed using ‘Summer Black’ grape berry as a material. The main results were as follows: A total of 11 transcripts involved in the primary metabolic pathways were significantly changed by the RR treatment. Metabolites such as sugars, organic acids, amino acids, starch, pectin, and cellulose were qualitatively and quantitatively analyzed along with their metabolic pathways. Sucrose synthase (*VIT_07s0005g00750*, *VIT_11s0016g00470*) and sucrose phosphate synthase (*VIT_18s0089g00410*) were inferred to play critical roles in the accumulation of starch, sucrose, glucose, and fructose, which was induced by the RR treatment. RR treatment also promoted the malic acid and tartaric acid accumulation in the young berry. In addition, the grape berries after the RR treatment tended to have lower pectin and cellulose content.

## 1. Introduction

Grape growth is characterized during the initial phase, when the berries are small, hard, and acidic. During the final phase, it becomes larger, softer, and sweeter due to the accumulation of a series of organoleptic compounds. Grape berries undergo a series of complex biochemical and physiological changes during growth, which can be divided into three distinct phases [1,2]. In the first phase, the berry size increases sigmoidally due to the cell division and expansion. In addition, organic acids (mainly tartaric and malic acids) are synthesized in the vacuoles and reach maximal concentrations at the end of this phase. On the contrary, the second phase is known as the lag phase, during which no increment of berry size occurs but sugars begin to accumulate. Afterwards, the veraison marks start, and this stage is characterized by the initiation of color development. In the third phase, the berry size continuously increases, the berry softens, and glucose and fructose increase rapidly. Furthermore, organic acids’ concentrations decrease. Besides, a large number of flavor metabolites and aromas are synthesized at the end of this phase [2,3].

Grape berries’ flavor depends mainly on the primary metabolites such as sugars, organic acids, amino acids, starch, pectin, and cellulose. The composition of metabolites varies from different natural environments and external stresses. Several studies deal with the interactions between environmental factors and the chemical compositions of berries [4]. For example, water stress significantly promotes favorable changes in grape berry and the accumulation of primary metabolites [5,6,7,8]. Climate change, temperature, salinity, and hormones significantly affect the final composition of the primary metabolites of grape berry in both the developmental and ripening stages [9,10,11,12,13,14,15,16].

Root restriction (RR) is a cultivation technique that controls the size of the shoot, optimizes the balance of vegetative growth and reproductive growth, and improves the efficiency of agricultural resources by restricting the root growth in a certain volume [17,18]. Recently, RR was found not only to increase the content of total and individual anthocyanins, but also to enrich the anthocyanin composition compared with control [19]. Most importantly, grape berry under RR treatment has a higher sugar content, and it was found that the acid invertase may be a key enzyme for promoting sugar accumulation [18,19,20]. However, there are few studies investigating the changes in the compositions and contents of other primary metabolites of grape berry following RR treatment during growth. However, plenty of physiological and molecular changes occur in the grape berry after RR treatment.

Thus, transcriptomics and primary metabolites analysis were conducted to explore the effects of RR treatment on ‘Summer Black’ grape berries in this study. Here, we identified the grape metabolic changes and molecular pathways triggered by RR treatment throughout the fruit’s developmental stages.

## 2. Materials and Methods

This study was carried out during the fruit season of 2013–2014 in a film greenhouse of Jinhua Academy of Agricultural Sciences (Zhejiang, China). Three-year-old ‘Summer Black’ (*V. vinifera* × *V. labrusca*) table grapes were used as the subjects, which were grown from cuttings obtained from grapevines in a common vineyard in Japan. The grapevine with the RR treatment was planted in isolation with the plastic film from the outside ground. The control group was planted with the same soil on the open ground. The same watering and fertilizer strategies were applied to the two groups. Grapevines were watered by a drip irrigation system to avoid different water deficits. According to the physiological and biochemical processes of grape, berries were collected in five different developmental stages at the same time: S1, fruitlet, 15d after full bloom (DAFB); S2, immature green, 28 DAFB; S3, before veraison (sugars start to accumulate and organic acids start to drop), 42 DAFB; S4, veraison, 53 DAFB; S5, fully ripe, 74 DAFB, respectively. About 10 clusters were randomly picked for each treatment at each sampling time. All samples were picked and selected to ensure consistent ripeness and no disease or mechanical damage. The fruits were cut into small pieces, and immediately frozen with liquid nitrogen. After being transported to the laboratory, the samples were stored at −80 °C for subsequent testing. Three biological replicates were set for each sample.

Sugars, organic acids, and amino acids: All samples were extracted and derivatized according to previously established procedures [21] with modifications. Approximately 0.1 g of berry powder was added with 1.4 mL of HPLC-grade methanol. The mixture was thoroughly vortexed and incubated at 70 °C for 15 min, then centrifuged at 11,000× *g* for 10 min. Then, 1.5 mL of millipore water containing 750 μL of HPLC grade chloroform was added to the supernatant and then spin down at 2200× *g* for 10 min. The upper phase of 100 μL was isolated, added with ribitol (0.2 mg/mL) (10 μL per 100 μL upper phases) as an internal standard, and dried down in a vacuum concentrator at room temperature. All samples were derivatized by adding 60 μL of methoxyamine HCl in pyridine (20 mg/mL), vortexed, and then incubated at 37 °C for 90 min. After that, 40 μL of MSTFA + 1% TMCS (Sigma-Aldrich, USA) were added, vortexed, and incubated at 37 °C for 30 min. The derivatized samples were analyzed using an Agilent 7890 GC coupled to a 5975 MSD scanner. A quantity of 1 μL of each sample was injected at a split ratio of 10:1, and the inlet and transfer line were held at 250 °C. Separation was achieved using a fused-silica capillary column (60 m × 0.25 mm i.d., 0.25 μm HB-5MS stationary phase) in the following temperature program: 100 °C for 1 min, ramped at 3 °C/min to 184 °C, increased to 190 °C at 0.5 °C/min, increased to 250 °C at 10 °C/min, held for 1 min, increased to 280 °C at 5 °C/min, and then held for 3 min. The flow rate was set as 1.0 mL/min.

Cellulose: Cellulose was extracted and measured according to the colorimetric method [22] with some modifications. Briefly, 100 mg of lyophilized berry powder was extracted with 5 mL of an acetic–nitric acid reagent (prepared by mixing 150 mL of 80% acetic acid and 15 mL of concentrated nitric acid) in a 100 °C water bath for 60 min. The precipitates were collected by centrifugation. All the non-cellulosic materials were removed by means of subsequent washing with distilled water and acetone. The residues were dried at 30 °C for 30 min, and then 9 mL of 67% H_2_SO_4_ (*v*/*v*) was added. The supernatant of 1 mL was obtained and distilled water was added to make a final volume of 50 mL. Afterwards, 1 mL of the diluted sample was mixed with 1 mL phenol and 5 mL H_2_SO_4_ and reacted at room temperature for 30 min. The absorbance of the reacted product was detected at 490 nm. 

Pectin: The pectin of lyophilized berry powder was extracted and measured according to a previously described method [23] with modifications. About 1 g of powder and 25 mL of 95% ethanol were heated in a boiling water bath for 30 min followed by cooling and centrifugation at 8000 rpm for 15 min. The supernatant was discarded. The precipitate was washed three times with 25 mL of 95% ethanol in order to ensure the removal of all non-pectin materials. The residues were extracted twice with 20 mL of distilled water in the water bath at 50 °C for 30 min, and then the supernatants were collected and transferred into a 100 mL volumetric flask and made up to volume with distilled water for the determination of water-soluble pectin. The remaining residues were extracted with 25 mL of sulfuric acid (0.5 mol/L) in a boiling water bath for 60 min. The supernatants were collected by centrifugation at 8000 rpm for 15 min and made up to 100 mL with distilled water for protopectin determination. The extract of each sample (1 mL) was mixed with 0.2 mL of 0.1% alcoholic carbazole and 6 mL of concentrated sulfuric acid. The mixtures were heated in a water bath at 85 °C for 10 min and cooled down for 15 min. The absorbance of the reaction products was immediately measured at 530 nm using the colorimetric method. Galacturonic acid was used as a standard. 

Starch: About 1 g of berry powder was transferred to a flask and incubated with 20 mL DMSO and 5 mL HCl (8 mol/L) at 60 °C for 30 min in a shaking water bath. Then, 50 mL of deionized water was added to the flask and the pH was adjusted to 4–5 using 5 mol/L NaOH. The solution was cooled down to room temperature and diluted to 100 mL using deionized water. The starch content was quantitated by means of the enzymatic method using a starch assay kit (Sigma–Aldrich, St. Louis, MO, USA). The starch content was measured at 340 nm.

Total RNAs were extracted following our previously published method [24], quantified using a Nanophotometer Pearl (Implen, Germany), and then used for RNA-seq and real-time PCR analysis. The cDNA libraries were constructed with three replicates using the TruSeqTM RNA sample preparation kit (Illumina, Inc., San Diego CA, USA). The RNA-Seq was carried out in Shanghai Majorbio Bio-pharm Biotechnology Co. (Shanghai, China), while the raw reads were obtained using the Illumina HiSeqTM 2000. Clean reads were obtained by removing the adapter and low-quality sequences using the software SeqPrep (https://github.com/jstjohn/SeqPrep, accessed on 20 December 2015) and aligned to the reference *Vitis vinifera* genome (http://www.genoscope.cns.fr/externe/Download/Projets/Projet_ML/data/, accessed on 22 December 2015) [25] using TopHat 2.0.13 software (http://tophat.cbcb.umd.edu/, accessed on 23 December 2015) [26] The RSeQC-2.3.2 program (http://code.google.com/p/rseqc/, accessed on 23 December 2015) was adopted to assess the saturation analysis, duplicate reads, and gene coverage analysis [27]. The Cufflinks program (http://cufflinks.cbcb.umd.edu/, accessed on 23 December 2015) was used to calculate the gene expression values. EdgeR software was used to analyze the differences in expression of each transcript in the two libraries [28,29,30]. The KEGG (Kyoto Encyclopedia of Genes and Genomes) function of the blast2go webtool was used to analyze the metabolic pathways.

All the raw sequence data were deposited at the NCBI Sequence Read Archive (SRA) with the following accession codes: SRX2234711/ SRR4408346, SRX2234711/ SRR4408347, SRX2234711/ SRR4408413, SRX2234711/ SRR4408414. Permission was obtained to collect the plant samples.

Real-time quantitative PCR was performed on a LightCycler 480 instrument (Roche, Basel, Switzerland) and analyses were carried out using the primers listed in Table 1 according to our previous method [31]. The GAPDH (Glyceraldehyde 3-phosphate dehydrogenase. NCBI accession number: CBI14856.3) gene was employed as the internal control for calculating the relative expression of the mRNA.

The results were presented as the mean ± standard error (SE) of at least three independent replicates. Absolute quantification of the individual compositions was conducted, except the soluble pectin and protopectin contents, as a standard. A SPSS version 16.0 statistical software package (IBM) was applied for data processing. The statistical significance of differences was determined by student t-test. The Origin 8.0 (Microcal Software) was applied for figures construction. 

## 3. Results

### 3.1. Differential Gene Expression in the Primary Metabolic Pathways

From the RNA-Seq results, significant changes were found in the expressions of 11 genes that were involved in sucrose synthesis and degradation, glycolysis, and the tricarboxylic acid cycle (TCA) metabolic pathways of the two treatments during different developmental stages in the primary metabolic pathways (Table 2). The differential gene expression occurred mainly at the earlier stages, especially in the fruitlet period, while no expression occurred in the ripening phase. 

### 3.2. Compounds in the Primary Metabolic Pathways

**Figure 1:**
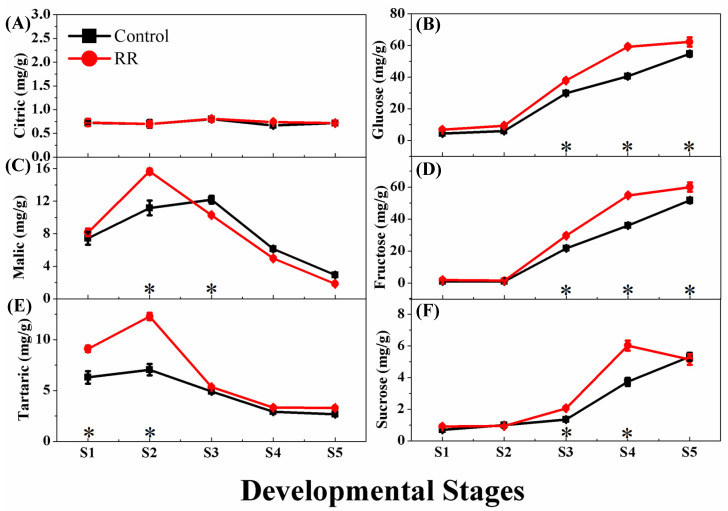
Effects of RR treatment on the content change of sugars and organic acids of the grape berry. (**A**) Citric; (**B**) glucose; (**C**) malic; (**D**) fructose; (**E**) tartaric; (**F**) sucrose. * indicates significant differences (*p* < 0.05, *n* = 3).

The quantitative analysis of the sugars and organic acids of grape berry was carried out at different developmental stages using GC-MS. The results showed that the predominant sugars were glucose and fructose, which accumulated sharply and rapidly at a 1:1 ratio before the veraison phase, and then increased slowly, reaching the peak at the ripening phase. RR treatment significantly increased the accumulation of glucose and fructose during the development and ripening stages. In the ripening phase, the glucose and fructose concentrations were 62 mg/g and 60 mg/g, respectively, in the RR treatment, and were significantly higher than the corresponding values of the control (54 mg/g and 52 mg/g, respectively). However, for sucrose, RR treatment did not increase its content at the maturity phase, but it could be promoted to reach the peak level earlier. Malic acid and tartaric acid were the dominant organic acids for grape berry, which accumulated at the earlier stages and then decreased during the developmental stage. RR treatment significantly promoted the accumulation of malic acid and tartaric acid in the young berries. However, the content of citric acid was stable during the whole development stage and was not affected by RR treatment (Figure 1).

**Figure 2:**
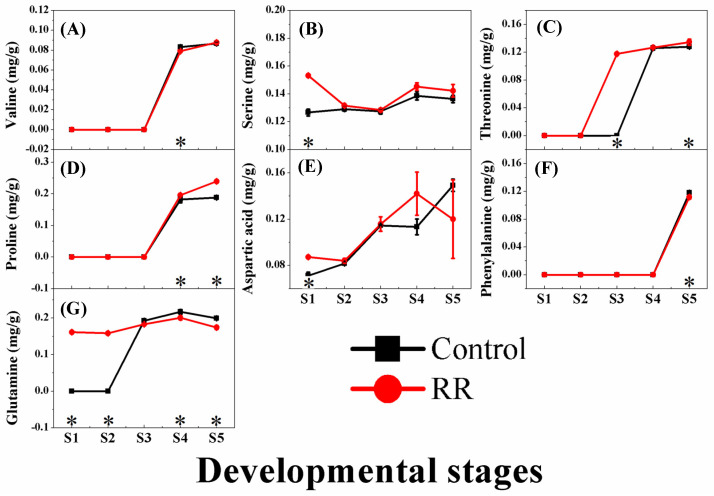
Effects of RR treatment on the content change of amino acids of grape berry. (**A**) Valine; (**B**) serine; (**C**) threonine; (**D**) proline; (**E**) aspartic acid; (**F**) phenylalanine; (**G**) glutamine. * indicates significant differences (*p* < 0.05, *n* = 3).

During the development and ripening stages, a total of seven amino acids (valine, serine, threonine, proline, aspartic acid, phenylalanine, and glutamine) were detected in the ‘Summer Black’ grape berries. The content of these amino acids showed variations in different stages of berry growth and development processes. In the earlier stages, RR treatment significantly promoted the accumulation of serine, aspartic acid, and glutamine, and most notably, could produce glutamine earlier. In the later stages, the contents of threonine and proline were increased by RR treatment. Between these, proline was particularly affected, with the content in the RR treatment being 26.32% higher than that in the control. The concentrations of valine, phenylalanine, and glutamine for the samples in the RR treatment were lower than those of the control in the last phase (Figure 2).

**Figure 3:**
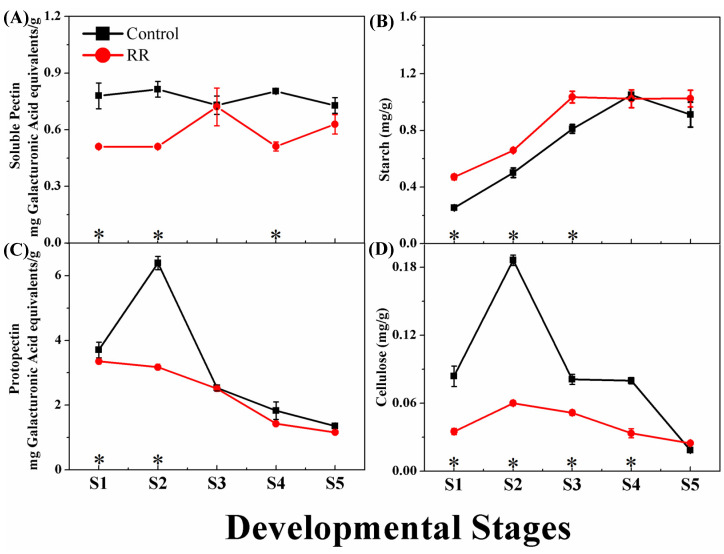
Effects of RR treatment on the content change of starch, pectin, and cellulose of grape berry. (**A**) Soluble pectin; (**B**) starch; (**C**) protopectin; (**D**) cellulose. * indicates significant differences (*p* < 0.05, *n* = 3).

In the present study, the starch content of grape berry showed an increasing trend during the development and ripening process. RR treatment induced starch accumulation before the veraison stages, but there was no significant difference between the two treatments at the S4 stage. In the ripening phase, the starch concentrations was 1.03 mg/g in the RR treatment, which was significantly higher than that of the control (0.91 mg/g). Pectin can be divided into protopectin and soluble pectin. The protopectin content of grape berry decreased during the development and ripening stages, but the soluble pectin content was relatively constant throughout the berry growth process. RR treatment significantly reduced the content of total pectin in the earlier stages, while the soluble pectin was reduced in the veraison phase. Our results showed that the cellulose content was increased and reached the peak before veraison, and then decreased continuously. RR treatment significantly reduced the cellulose content during the all developmental stages except the ripening stage (Figure 3).

### 3.3. Validation of Different Gene Expression Using qRT-PCR

**Figure 4:**
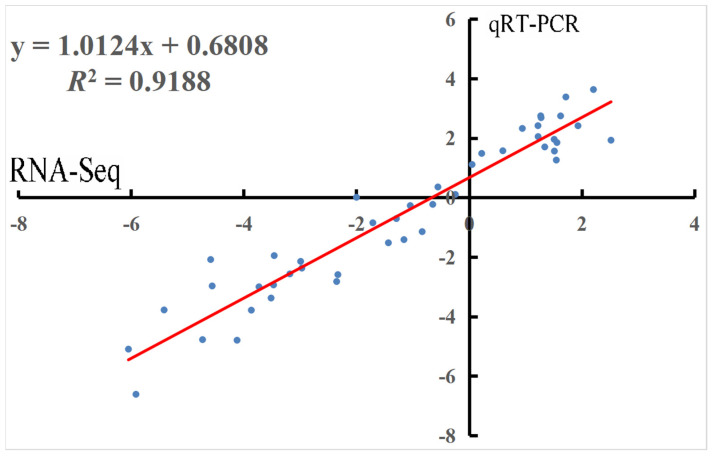
qRT-PCR validation of the transcripts between two treatments of grape berries during development and ripening..Correlation of fold change was analyzed by RNA-Seq (x axis) and the data were obtained using qRT-PCR (y axis).

Fifty transcripts involved in the primary metabolic pathways were randomly selected for qRT-PCR detection. These randomly selected genes (*VIT_01s0010g02460*, *VIT_06s0004g02620*, *VIT_07s0005g00750*, *VIT_13s0019g04460*, and *VIT_13s0074g00390*) were upregulated, downregulated, and unaffected by RR treatment compared to control. Linear regression analysis showed an overall determination coefficient of 0.9188 (Figure 4), indicating that the qRT-PCR expression profiles were in agreement with the RNA-Seq values. The determination coefficient result suggested the reliability of the RNA-Seq data.

## 4. Discussion

The sequence reads of 150 Gb were generated from all samples. The expression of 29,971 transcripts was detected after aligning the sequence reads on the grape reference genome. Among these, the 1264, 313, 141, 246, and 19 transcripts were significantly changed between two treatments in different developmental stages. A relatively large number of differential transcripts were found in the earlier stages, which were linked with photosynthetic capacity and primary metabolism, whereas the differential transcripts in the later stages were mainly linked with secondary metabolism [31,32]. Table grape quality, sweetness, and low acidity are the most important parameters in the market. Our results were in agreement with previous studies [18], which showed that RR can improve fruit quality by promoting the accumulation of sugars in grape berries. In addition, other primary metabolites such as organic acids, amino acids, starch, pectin, cellulose, etc. that affect grape flavor were also investigated by transcriptomic analysis. 

**Figure 5:**
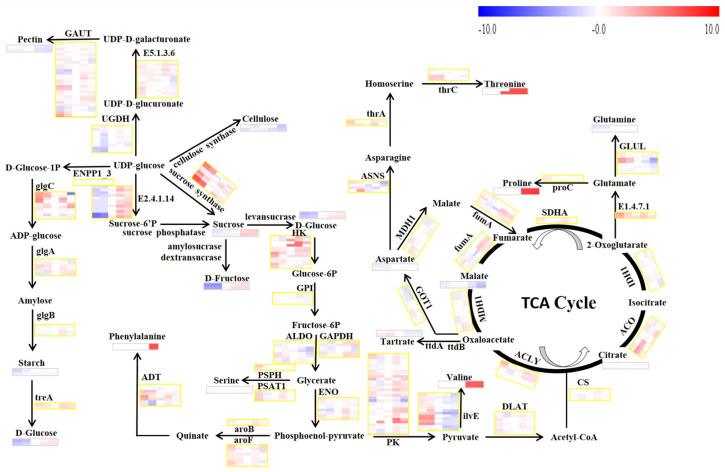
Effects of RR treatment on the primary metabolic pathways in grape berry. Boxes corresponding with the berry development process, from left to right, were obtained by using the MultiExperiment Viewer software. The dataset was normalized to the values in control treatment at the stages before veraison via log_2_ transformation. The relative expression changes at the RR treatment and other stages in relation to the stages before veraison in control treatment were expressed as log_2_ fold changes. The upper set of boxes was for the control treatment and the lower set was for the RR treatment. thrA, bifunctional aspartokinase/homoserine dehydrogenase 1; UGDH, UDP-glucose 6-dehydrogenase; MDH1, malate dehydrogenase; IDH1, isocitrate dehydrogenase; GAPDH, glyceraldehyde 3-phosphate dehydrogenase; SDHA, succinate dehydrogenase flavoprotein subunit; E1.4.7.1, glutamate synthase; proC, pyrroline-5-carboxylate reductase; DLAT, dihydrolipoamide acetyltransferase; CS, citrate synthase; ACLY, ATP citrate lyase; E2.4.1.14, sucrose phosphate synthase; glgB, 1,4-alpha-glucan branching enzyme; glgA, starch synthase; GAUT, alpha-1,4-galacturonosyltransferase; aroF, 3-deoxy-7-phosphoheptulonate synthase; GOT1, aspartate aminotransferase; ilvE, branched-chain amino acid aminotransferase; serC, phosphoserine aminotransferase; HK, hexokinase; PK, pyruvate kinase; glgC, glucose-1-phosphate adenylyltransferase; serB, phosphoserine phosphatase; treA, alpha-trehalase; ENPP1_3, ectonucleotide pyrophosphatase/phosphodiesterase family member 1/3; ALDO, fructose-bisphosphate aldolase; fumC, fumarate hydratase; ACO, aconitate hydratase; ENO, enolase; ADT, arogenate dehydratase; thrC, threonine synthase; aroB, 3-dehydroquinate synthase; E5.1.3.6, UDP-glucuronate 4-epimerase; GPI, glucose-6-phosphate isomerase; glnA, glutamine synthetase; ASNS, asparagine synthase.

The primary metabolites studied in our experiments were involved in the pathways of primary carbohydrate metabolism, such as sucrose synthesis and degradation, glycolysis, and the TCA cycle [4]. To facilitate the visualization and global analysis of the differences in gene expression and primary metabolites between the RR and control treatments during the development and ripening stage of grape berry, the gene expression levels of both groups were normalized to that of the S3 stage in the control group (Figure 5) 

Sugars, especially glucose, fructose, and sucrose, determine the sweetness of grapes’ berries. Sucrose is transported from the leaves through the phloem, and then is hydrolyzed to fructose and glucose at a suitable pH level. The sucrose content could be influenced by a disproportionate ratio of import to consumption during the development and ripening of the grape berry [33,34,35]. Sugar accumulation in the berries depends on the activity of sugar-metabolizing enzymes during the developmental and ripening stages. The enzymes include invertase, sucrose synthase, and sucrose phosphate synthase [36]. Sugar concentration is strongly affected by environmental stresses and cultivation management [37]. Previous studies showed that RR treatment reduced the photosynthetic rate and led to the distribution of more dry material into berries [20]. It also increased the number of plasmodesmata between sieve elements and companion cells, between sieve element/companion cell complexes, and between phloem parenchyma cells [38]. These variations might be due to the increase in sucrose transport into the berries. Meanwhile, the change of acid invertase activity showed a similar trend to the change of total sugar content. Thus, it can be deduced that the acid invertase may be the critical enzyme in the promotion of glucose and fructose accumulation caused by RR treatment [18]. Uridine diphosphate glucose (UDP-glucose), a nucleotide sugar, is the precursor of sucrose, starch and cell-wall polysaccharides, and sucrose synthase. UDP-glucose plays an important role in the biosynthetic pathways of these metabolites [33]. In our study, we observed that one gene (*VIT_07s0005g00750*) coding for the sucrose synthase was up-regulated, but another gene (*VIT_11s0016g00470*) coding for the sucrose synthase was down-regulated at the S1 stage as a result of the RR treatment. It is interesting to note that one gene (*VIT_18s0089g00410*) coding for the sucrose phosphate synthase was up-regulated at the S4 stage by RR treatment, which was in agreement with the increase in sugar contents. Thus, this gene might play a critical role in the accumulation of sugars (Table 2 and Figure 5). 

In grape berries, polysaccharide is the most critical substance that affects the softening of the grape, the contents of which decrease during ripening [39]. Pectin and cellulose are considered to constitute a large proportion of the polysaccharide components. Pectin can be classified as a protopectin and a soluble pectin. Protopectin was found to be gradually converted into soluble pectin in the process of grape berry development and ripening [40]. The results of the changes of polysaccharide contents in our studies were similar to the results of previous studies [39]. Cellulose and pectin biosynthesis share the same precursor as sucrose, and compete with each other [41,42]. In our study, sugars accumulated and their contents were increased as a result of RR treatment. This indicates that the content of both cellulose and pectin was decreased by RR treatment during the development stage. In addition, one gene (*VIT_01s0011g00690*) coding for UDP-glucose 6-dehydrogenase was up-regulated at the S1 stage by RR treatment, and this gene was a putatively negative regulator of the biosynthesis of pectin in the grape berry. Starch is generally regarded as the major storage carbohydrate in higher plants [43]. Adenosine diphosphate glucose (ADP-glucose) is the substrate for starch biosynthesis, whereas the starch synthase (glgA) 1,4-alpha-glucan branching enzyme (glgB) and alpha-trehalase (treA) played critical roles in the starch synthesis and degradation pathways (Figure 5). However, the activity of these enzymes is complex in storage organs such as fruits [33]. We observed that one gene (*VIT_02s0025g02170*) coding for alpha-trehalase in the starch degradation pathway was down-regulated byRR treatment at the S1 stage; this gene putatively regulates the content of starch [44].

Respiration of sugars via glycolysis and the TCA cycle provides energy (ATP), NAD(P)H and precursors for the biosynthesis of organic acids, amino acids, and many secondary metabolites [4]. Malic acid is the main organic acid, which accumulates from fruitlets until veraison in the vacuoles of grape berry cells. In the post-veraison stages, malic acid is a vital source of carbon for different pathways of the primary and secondary metabolism, including the TCA cycle, ethanol fermentation and gluconeogenesis, amino acid interconversions, and the production of secondary compounds such as anthocyanins and flavonols [34,45]. Tartaric acid is another chief organic acid in grape berries, which accumulates before veraison and then shows a decline until harvest. It was found to be produced from the oxaloacetate and ascorbate degradation pathway [46,47]. We found increased contents of malic acid and tartaric acid at the earlier stages; this was due to the increased sugar content in the upstream metabolic pathway caused by RR treatment. However, at the veraison stage, RR treatment could promote the consumption of malic acid. Citric is an intermediate of the TCA cycle and was not affected by RR treatment for grape berry.

The expression of genes involved in amino acids’ metabolic pathways was differentially affected by RR treatment in our experiment. Proline is one of the most abundant amino acids in grape berry, and is accumulated to high levels at the later stages of fruit ripening [46]. Proline content was significantly increased under water deficit and salinity stresses [43]. A previous study showed that RR can increase the proline content of mango trees [48]. Our results showed that proline content increased at the veraison and ripening stages in both treatments. In addition, there were significantly higher proline concentrations for the samples from the RR treatment compared with the control group (*p* < 0.01). The glutamate content in grape berry was too low to provide reliable quantitative data in the GC-MS chromatograms. However, it plays an important role in the proline and glutamine biosynthesis as the common intermediate. RR treatment increased the content of glutamine at the earlier stages of grape growth, but reduced the glutamine concentrations at the later stages of grape growth. This was because of the accumulation of proline occurred at the same time. The effect of RR treatment on the expression of genes involved in the metabolic pathways of glutamate to proline or glutamine was not significant. Phenylalanine is a precursor of the phenolic compounds that accumulate at maturity, and 3-deoxy-7-phosphoheptulonate synthase is putatively involved in phenylalanine biosynthesis [33]. Therefore, it is not surprising that phenylalanine content was decreased by RR treatment at maturity, but the gene (*VIT_00s0391g00070*) coding for 3-deoxy-7-phosphoheptulonate synthase showed higher expression than that of the control. Meanwhile, it is interesting to note that the genes that were likely encoding for glycolysis and TCA cycle enzymes, such as glyceraldehyde 3-phosphate dehydrogenase (*VIT_01s0010g02460*), enolase (*VIT_16s0022g01770*), branched-chain amino acid aminotransferase (*VIT_18s0001g08420*), aconitate hydratase (*VIT_12s0059g02150*), and asparagine synthase (*VIT_06s0004g06830*), were involved in the organic acids’ and amino acids’ metabolic pathways.

## 5. Conclusions

In summary, this work firstly described a comprehensive transcriptomic analysis of the effects of RR on the primary metabolites during the development and ripening of grape berry. The combined analysis of genes and compounds contributed to exploring the general effects of the RR treatment on the primary metabolisms during the whole fruit growth process. In general, RR treatment induced the accumulation of malic, tartaric, and starch in the earlier stages. In addition, RR treatment also increased the content of glucose, fructose, sucrose, and proline in the later stages. In contrast, RR treatment decreased the concentrations of soluble pectin, protopectin, and cellulose at different developmental stages. The changes of these primary metabolites might be attributed to the changes of sucrose synthase and sucrose phosphate synthase, which were induced by RR treatment. The findings of this study would be helpful in increasing our understanding of grape berry’s responses to RR treatment.

## Figures and Tables

**Table 1:** Primers for real-time PCR.

Gene	Forward Primer (5’ to 3’)	Reverse Primer (5’ to 3’)
*GAPDH*	TGGAGCTGAATTTGTTGT	GTGGAGTTCTGGCTTGTA
*VIT_01s0010g02460*	ACTACCAACTGTCTTGCTCCTCTG	AGTAAGGTCCACGACTGAAACATC
*VIT_06s0004g02620*	CCTCAACGCCAACATTAG	GCCAAACCAGACCCTACT
*VIT_07s0005g00750*	GATGTAGGGCGGCAGAAACT	GAACAGCAATAGCCACAAAAGG
*VIT_13s0019g04460*	ACTGCTGTTGGGTCTGGC	GAGGGCGTATCGGTCTTG
*VIT_13s0074g00390*	AAACACCCTCCCACCTAC	TATCCTTCGCCCTACTCC

**Table 2:** Differentially expressed genes in the primary metabolic pathways under RR treatment during grape development (FDR < 0.05 and Log_2_|FC| > 1). FDR, false discovery rate; FC, fold change.

Gene ID	Log_2_FC					Functional Annotation
	S1RR/ S1Control	S2RR/ S2Control	S3RR/ S3Control	S4RR/ S4Control	S5RR/ S5Control	
*VIT_01s0011g00690*	1.44	-	-	-	-	UDP-glucose 6-dehydrogenase
*VIT_01s0010g02460*	1.50	1.95	-	-	-	glyceraldehyde 3-phosphate dehydrogenase
*VIT_07s0005g00750*	1.69	-	-	-	-	sucrose synthase
*VIT_11s0016g00470*	−1.33	-	-	-	-	sucrose synthase
*VIT_18s0089g00410*	-	-	-	1.55	-	sucrose-phosphate synthase
*VIT_00s0391g00070*	-	-	-	1.30	-	3-deoxy-7-phosphoheptulonate synthase
*VIT_18s0001g08420*	1.93	-	−1.60	-	-	branched-chain amino acid aminotransferase
*VIT_02s0025g02170*	−1.17	-	-	-	-	alpha-trehalase
*VIT_12s0059g02150*	−1.03	-	-	-	-	aconitate hydratase
*VIT_16s0022g01770*	1.30	-	-	-	-	enolase
*VIT_06s0004g06830*	1.32	2.25	-	-	-	asparagine synthase

## Data Availability

Data are contained within the article.

## References

[1] Niculcea M., Martinez-Lapuente L., Guadalupe Z., Sanchez-Diaz M., Morales F., Ayestaran B., Antolin M.C. (2013). Effects of Water-Deficit Irrigation on Hormonal Content and Nitrogen Compounds in Developing Berries of *Vitis vinifera* L. cv. Tempranillo. J. Plant Growth Regul..

[2] Deluc L.G., Grimplet J., Wheatley M.D., Tillett R.L., Quilici D.R., Osborne C., Schooley D.A., Schlauch K.A., Cushman J.C., Cramer G.R. (2007). Transcriptomic and metabolite analyses of Cabernet Sauvignon grape berry development. BMC Genom..

[3] Zoccatelli G., Zenoni S., Savoi S., Dal S.S., Tononi P., Zandona V., Dal C.A., Guantieri V., Pezzotti M., Tornielli G.B. (2013). Skin pectin metabolism during the postharvest dehydration of berries from three distinct grapevine cultivars. Aust. J. Grape Wine Res..

[4] Dai Z.W., Leon C., Feil R., Lunn J.E., Delrot S., Gomes E. (2013). Metabolic profiling reveals coordinated switches in primary carbohydrate metabolism in grape berry (*Vitis vinifera* L.), a non-climacteric fleshy fruit. J. Exp. Bot..

[5] Deluc L.G., Quilici D.R., Decendit A., Grimplet J., Wheatley M.D., Schlauch K.A., Merillon J.M., Cushman J.C., Cramer G.R. (2009). Water deficit alters differentially metabolic pathways affecting important flavor and quality traits in grape berries of Cabernet Sauvignon and Chardonnay. BMC Genom..

[6] Castellarin S.D., Pfeiffer A., Sivilotti P., Degan M., Peterlunger E., Di G.G. (2007). Transcriptional regulation of anthocyanin biosynthesis in ripening fruits of grapevine under seasonal water deficit. Plant Cell Environ..

[7] Berdeja M., Nicolas P., Kappel C., Dai Z.W., Hilbert G., Peccoux A., Lafontaine M., Ollat N., Gomes E., Delrot S. (2015). Water limitation and rootstock genotype interact to alter grape berry metabolism through transcriptome reprogramming. Hortic. Res..

[8] Kyraleou M., Koundouras S., Kallithraka S., Nikolaos T., Niki P., Yorgos K. (2015). Effect of irrigation regime on anthocyanin content and antioxidant activity of *Vitis vinifera* L. cv. Syrah grapes under semiarid conditions. J. Sci. Food Agric..

[9] Lee H., Patrick R.R., Makihiko I., Anderson V., Shepard M., Rebecca S., Gary T., Lu Z., Marquet P.A., Hijmans R.J. (2013). Climate change, wine, and conservation. Proc. Natl. Acad. Sci. USA.

[10] Cramer G.R., Ergul A., Grimplet J., Tillett R.L., Tattersall E.A.R., Bohlman M.C., Vincent D., Sonderegger J., Evans J., Osborne C. (2007). Water and salinity stress in grapevines: Early and late changes in transcript and metabolite profiles. Funct. Integr. Genom..

[11] Berli F.J., Fanzone M., Piccoli P., Bottini R. (2011). Solar UV-B and ABA Are Involved in Phenol Metabolism of *Vitis vinifera* L. Increasing Biosynthesis of Berry Skin Polyphenols. J. Agric. Food Chem..

[12] Carbonell-Bejerano P., Maria E.S., Torres-Perez R., Royo C., Lijavetzky D., Bravo G., Aguirreolea J., Sanchez-Diaz M., Antolin M.C., Martinez-Zapater J.M. (2013). Thermotolerance Responses in Ripening Berries of *Vitis vinifera* L. cv Muscat Hamburg. Plant Cell Physiol..

[13] Zhang C., Jia H., Wu W.M., Wang X.C., Fang J.G., Wang C. (2015). Functional conservation analysis and expression modes of grape anthocyanin synthesis genes responsive to low temperature stress. Gene.

[14] Mierziak J., Kostyn K., Kulma A. (2014). Flavonoids as Important Molecules of Plant Interactions with the Environment. Molecules.

[15] Shinomiya R., Fujishima H., Muramoto K., Shiraishi M. (2015). Impact of temperature and sunlight on the skin coloration of the ‘Kyoho’ table grape. Sci. Hortic..

[16] Alessandra F., Claudio L. (2014). Abiotic stress effects on grapevine (*Vitis vinifera* L.): Focus on abscisic acid-mediated consequences on secondary metabolism and berry quality. Environ. Exp. Bot..

[17] Yang T.Y., Zhu L.N., Wang S.P., Gu W.J., Huang D.F., Xu W.P., Jiang A.L., Li S.C. (2007). Nitrate uptake kinetics of grapevine under root restriction. Sci. Hortic..

[18] Xie Z.S., Li B., Forney C.F., Xu W.P., Wang S.P. (2009). Changes in sugar content and relative enzyme activity in grape berry in response to root restriction. Sci. Hortic..

[19] Wang B., He J.J., Bai Y., Yu X.M., Li J.F., Zhang C.X., Xu W.P., Bai X.J., Cao X.J., Wang S.P. (2013). Root restriction affected anthocyanin composition and up-regulated the transcription of their biosynthetic genes during berry development in ‘Summer Black’ grape. Acta Physiol. Plant..

[20] Wang B., He J.J., Duan C.Q., Yu X.M., Zhu L.N., Xie Z.S., Zhang C.X., Xu W.P., Wang S.P. (2012). Root restriction affects anthocyanin accumulation and composition in berry skin of ‘Kyoho’ grape (*Vitis vinifera* L. x *Vitis labrusca* L.) during ripening. Sci. Hortic..

[21] Broeckling C.D., Huhman D.V., Farag M.A., Smith J.T., May G.D., Mendes P., Dixon R.A., Sumner L.W. (2005). Metabolic profiling of Medicago truncatula cell cultures reveals the effects of biotic and abiotic elicitors on metabolism. J. Exp. Bot..

[22] Li M.Y., Bahn S.C., Guo L., Musgrave W., Berg H., Welti R., Wang X.M. (2011). Patatin-Related Phospholipase pPLAIII beta-Induced Changes in Lipid Metabolism Alter Cellulose Content and Cell Elongation in Arabidopsis. Plant Cell.

[23] Dietz J.H., Rouse A.H. (1952). Evaluation of a Rapid Method for Estimating Pectic Substances in Citrus Juices. Food Technol..

[24] Shan L.L., Li X., Wang P., Cai C., Zhang B., Sun C.D., Zhang W.S., Xu C.J., Ferguson I., Chen K.S. (2008). Characterization of cDNAs associated with lignification and their expression profiles in loquat fruit with different lignin accumulation. Planta.

[25] Jaillon O., Aury J.M., Noel B., Policriti A., Clepet C., Casagrande A., Choisne N., Aubourg S., Vitulo N., Jubin C. (2007). The grapevine genome sequence suggests ancestral hexaploidization in major angiosperm phyla. Nature.

[26] Trapnell C., Pachter L., Salzberg S.L. (2009). TopHat: Discovering splice junctions with RNA-Seq. Bioinformatics.

[27] Wang L.G., Wang S.Q., Li W. (2012). RSeQC: Quality control of RNA-seq experiments. Bioinformatics.

[28] Li B., Dewey C.N. (2011). RSEM: Accurate transcript quantification from RNA-Seq data with or without a reference genome. BMC Bioinform..

[29] Robinson M.D., McCarthy D.J., Smyth G.K. (2010). edgeR: A Bioconductor package for differential expression analysis of digital gene expression data. Bioinformatics.

[30] Tang H.B., Wang X.Y., Bowers J.E., Ming R., Alam M., Paterson A.H. (2008). Unraveling ancient hexaploidy through multiply-aligned angiosperm gene maps. Genome Res..

[31] Leng F., Cao J.P., Ge Z.W., Wang Y., Zhao C.N., Wang S.P., Li X., Zhang Y.L., Sun C.D. (2020). Transcriptomic Analysis of Root Restriction Effects on Phenolic Metabolites during Grape Berry Development and Ripening. J. Agric. Food Chem..

[32] Leng F., Lin Q., Wu D., Wang S.P., Wang D.L., Sun C.D. (2016). Comparative Transcriptomic Analysis of Grape Berry in Response to Root Restriction during Developmental Stages. Molecules.

[33] Agudelo-Romero P., Erban A., Sousa L., Pais M.S., Kopka J., Fortes A.M. (2013). Search for Transcriptional and Metabolic Markers of Grape Pre-Ripening and Ripening and Insights into Specific Aroma Development in Three Portuguese Cultivars. PLoS ONE.

[34] Sweetman C., Deluc L.G., Cramer G.R., Ford C.M., Soole K.L. (2009). Regulation of malate metabolism in grape berry and other developing fruits. Phytochemistry.

[35] Wang L., Sun X.L., Weiszmann J., Weckwerth W. (2017). System-Level and Granger Network Analysis of Integrated Proteomic and Metabolomic Dynamics Identifies Key Points of Grape Berry Development at the Interface of Primary and Secondary Metabolism. Front. Plant Sci..

[36] Zhu S.M., Liang Y.L., An X.J., Kong F.C., Gao D.K., Yin H.F. (2017). Changes in sugar content and related enzyme activities in table grape (*Vitis vinifera* L.) in response to foliar selenium fertilizer. J. Sci. Food Agric..

[37] Dai Z.W., Ollat N., Gomes E., Decroocq S., Tandonnet J.P., Bordenave L., Pieri P., Hilbert G., Kappel C., van Leeuwen C. (2011). Ecophysiological, Genetic, and Molecular Causes of Variation in Grape Berry Weight and Composition: A Review. Am. J. Enol. Vitic..

[38] Xie Z.S., Forney C.F., Xu W.P., Wang S.P. (2009). Effects of Root Restriction on Ultrastructure of Phloem Tissues in Grape Berry. Hortscience.

[39] Wang D.D., Yeats T.H., Uluisik S., Rose J.K.C., Seymour G.B. (2018). Fruit Softening: Revisiting the Role of Pectin. Trends Plant Sci..

[40] Fasoli M., Dell’Anna R., Dal S.S., Balestrini R., Sanson A., Pezzotti M., Monti F., Zenoni S. (2016). Pectins, Hemicelluloses and Celluloses Show Specific Dynamics in the Internal and External Surfaces of Grape Berry Skin During Ripening. Plant Cell Physiol..

[41] Zogaj X., Nimtz M., Rohde M., Bokranz W., Romling U. (2001). The multicellular morphotypes of Salmonella typhimurium and Escherichia coli produce cellulose as the second component of the extracellular matrix. Mol. Microbiol..

[42] Molhoj M., Verma R., Reiter W.D. (2004). The biosynthesis of D-galacturonate in plants. Functional cloning and characterization of a membrane-anchored UDP-D-Glucuronate 4-epimerase from Arabidopsis. Plant Physiol..

[43] Somkuwar R.G., Bahetwar A., Khan I., Satisha J., Ramteke S.D., Itroutwar P., Bhongale A., Oulkar D. (2014). Changes in growth, photosynthetic activities, biochemical parameters and amino acid profile of Thompson Seedless grapes (*Vitis vinifera* L.). J. Environ. Biol..

[44] Fortes A.M., Agudelo-Romero P., Silva M.S., Ali K., Sousa L., Maltese F., Choi Y.H., Grimplet J., Martinez-Zapater J.M., Verpoorte R. (2011). Transcript and metabolite analysis in Trincadeira cultivar reveals novel information regarding the dynamics of grape ripening. BMC Plant Biol..

[45] Martinez-Esteso M.J., Selles-Marchart S., Lijavetzky D., Pedreno M.A., Bru-Martinez R. (2011). A DIGE-based quantitative proteomic analysis of grape berry flesh development and ripening reveals key events in sugar and organic acid metabolism. J. Exp. Bot..

[46] Grimplet J., Wheatley M.D., Jouira H.B., Deluc L.G., Cramer G.R., Cushman J.C. (2009). Proteomic and selected metabolite analysis of grape berry tissues under well-watered and water-deficit stress conditions. Proteomics.

[47] Munoz-Robredo P., Robledo P., Manriquez D., Molina R., Defilippi B.G. (2011). Characterization of Sugars and Organic Acids in Commercial Varieties of Table Grapes. Chil. J. Agric. Res..

[48] Zaharah S.S., Razi I.M. (2009). Growth, stomata aperture, biochemical changes and branch anatomy in mango (*Mangifera indica*) cv. Chokanan in response to root restriction and water stress. Sci. Hortic..

